# Functional Properties of Amino Acid Side Chains as Biomarkers of Extraterrestrial Life

**DOI:** 10.1089/ast.2018.1868

**Published:** 2018-10-26

**Authors:** Christos D. Georgiou

**Affiliations:** Department of Biology, University of Patras, Patras, Greece.

**Keywords:** Catalysis, Hydrolysis, Amino acids, Side chains, Terminal groups

## Abstract

The present study proposes to search our solar system (Mars, Enceladus, Europa) for patterns of organic molecules that are universally associated with biological functions and structures. The functions are primarily catalytic because life could only have originated within volume/space-constrained compartments containing chemical reactions catalyzed by certain polymers. The proposed molecular structures are specific groups in the side chains of amino acids with the highest catalytic propensities related to life on Earth, that is, those that most frequently participate as key catalytic groups in the active sites of enzymes such as *imidazole, thiol, guanidinium, amide,* and *carboxyl*. Alternatively, these or other catalytic groups can be searched for on non-amino-acid organic molecules, which can be tested for certain hydrolytic catalytic activities. The first scenario assumes that life may have originated in a similar manner as the terrestrial set of α*-*amino acids, while the second scenario does not set such a requirement. From the catalytic propensity perspective proposed in the first scenario, life must have invented amino acids with high catalytic propensity (His, Cys, Arg) in order to overcome, and be complemented by, the low catalytic propensity of the initially available abiogenic amino acids. The abiogenic and the metabolically invented amino acids with the lowest catalytic propensity can also serve as markers of extraterrestrial life when searching for patterns on the basis of the following functional propensities related to protein secondary/quaternary structure: (1) amino acids that are able to form α-helical intramembrane peptide domains, which can serve as primitive transporters in protocell membrane bilayers and catalysts of simple biochemical reactions; (2) amino acids that tend to accumulate in extremophile proteins of Earth and possibly extraterrestrial life. The catalytic/structural functional propensity approach offers a new perspective in the search for extraterrestrial life and could help unify previous amino acid–based approaches.

## 1. Amino Acids as Biomarkers of Extraterrestrial Life: Previous Approaches

Why should we search for amino acids as biomarkers in life-detection missions to other planetary objects? The simplest answers are that they are unique constituents of terrestrial life and some are found in meteorites. Reassessment of prebiotic organic synthesis in neutral planetary atmospheres dominated by N_2_, H_2_O, and CO_2_ has shown that the main amino acids produced are Ser, Glu, Gly, Ala, and Asp (along with traces of α-aminoisobutyric acid, γ-aminobutyric acid, and β-alanine), which supports the heterotrophic hypothesis of the origin of life (Cleaves *et al.,* 2008). Moreover, finding cyanide compounds (CH_3_CN, HC_3_N and HCN) in protoplanetary disks such as the young star MWC 480 is of special interest because of its cometlike composition. This supports the idea that comets once seeded early Earth with the water and organics needed for life to originate, as well as the role of C-N bonds for abiotic amino acid synthesis (Öberg *et al.,* 2015). Other supporting evidence comes from the discovery that the Kiliani-Fischer-type reductive homologation of hydrogen cyanide and some of its derivatives are the source of the precursors of amino acids (ribonucleotides and lipids as well) and that all the cellular subsystems of life could have arisen simultaneously through common prebiotic chemistry (Patel *et al.,* 2015). The heterotrophic appearance of life prompts remote sensing approaches for its detection even in exoplanets. A polarimetry-based remote-sensing method has been proposed for the detection and identification of life in distant worlds by linear polarization signals from biomolecules that can capture photons of particular wavelengths and can be distinguishable from mineral components of sands and rocks (Berdyugina *et al.,* 2016). However, it is questionable whether the polarization spectra calibrated from Earth plant pigments (such as chlorophyll, carotenoids, and others) would also be produced by the structurally unknown pigments in the surface, oceans, and clouds of exoplanets that are inhabited by extraterrestrial life. Abiogenic physicochemical processes involving carbon compounds have long been used to distinguish biological processes by stable carbon isotope fractionation. Since acetylene has been detected in Titan's upper atmosphere, and may also originate within Enceladus by thermal processes, it was proposed that stable carbon isotope fractionation (^12^C/^13^C) due to biotic acetylene fermentation could be used for life detection in hydrocarbon-rich volatiles of icy planet(oid)s (Miller *et al.,* 2015).

### 1.1. Abiogenic versus biogenic distributions of amino acids

More than 80 amino acids (α-, β-, γ-, and δ*-*type, ranging from C2 to C9) have been identified in carbonaceous meteorites (Pizzarello *et al.,* 2006; Pizzarello and Holmes, 2009; Pizzarello and Shock, 2010; Burton *et al.,* 2012b; Cobb and Pudritz, 2014; Elsila *et al.,* 2016). In thermally altered carbonaceous meteorites (type CV carbonaceous chondrites), in contrast, small, straight-chain, amine terminal (*n-omega*-amino) group-carrying amino acids predominate, which is not consistent with Strecker formation (Burton *et al.,* 2012a). Among α-amino acids, Gly, Ala, Val, Leu, Ile, Thr, Ser, Asp, Glu, and Pro are present in most meteorites (Cobb and Pudritz, 2014). Lys, Phe, and Tyr are considered to be solely the products of metabolism but have been detected in the CM2, CR2 meteorites (Pizzarello and Holmes, 2009), and this report was included in a review article (Cobb and Pudritz, 2014). The presence of Phe and Tyr in meteoritic organics would be highly significant and needs to be reproduced because their terrestrial concentrations are comparable (Moura *et al.,* 2013). Thus, possible contamination has not been ruled out.

It has been suggested that some of these amino acids could possibly have been inherited by extraterrestrial life in our solar system under the Second Genesis hypothesis (Smith and McKay, 2005). On the other hand, if the additional α-amino acids found in Earth life evolved (were invented) because they were essential, then they would presumably have been necessary components of extraterrestrial life biochemistry as well (Davila and McKay, 2014), which suggests that the first forms of life did not require all 20 biological amino acids. This logic also reduces the number of the initially required nucleobases to three (and the number of bases in a codon), which are most likely adenine, guanine, and uracil. All these are found in meteorites (Stoks and Schwartz, 1979, 1981; Shimoyama *et al.,* 1990). Nonetheless, cytosine, thymine, and uracil can be produced abiogenically via UV photoprocessing of pyrimidine in H_2_O-rich ice mixtures that contain NH_3_ or CH_4_ (Materese *et al.,* 2017). Thymine (found in DNA) is by far the lowest yield among the nucleobases synthesized in prebiotic simulations (Bera *et al.,* 2016), which corroborates with its absence in 11 different carbonaceous chondrites from groups CI, CM, and CR (Callahan *et al.,* 2011) and supports the hypothesis that RNA precedes DNA in the origin of Earth life. However, if such an invention was an outcome mostly of chance, we might then anticipate that an independent origin of life would rely on a different biochemical foundation. A selected set of amino acids by itself would not determine the biochemical framework for extraterrestrial life, because other macromolecules could have assumed catalytic roles in alien biochemistry.

Attempts have been made to use biochemical thermodynamics and the role of chance to guess which possible sets of amino acids alien life may harbor (McKay *et al.,* 2015). One approach is to consider whether natural selection would have favored a certain set of amino acids. This possibility was evaluated by (i) using the meteoritic amino acids to draw plausible alternative sets that could have been established at random; (ii) quantifying size, charge, and hydrophobicity for these amino acids; (iii) quantifying the “coverage” (“breadth and evenness of distribution”) for a given amino acid set; and (iv) calculating the expectations of a random alphabet of amino acids. It was concluded that the set of 20 amino acids used by terrestrial life cannot be explained by chance alone when viewed in terms of size, charge, and hydrophobicity (Philip and Freeland, 2011). Another analysis has suggested a role for thermodynamics in determining the order by which amino acids entered the standard alphabet. Meteoritic amino acids are the least thermodynamically costly to form; thus, they were most abundant before life arose (Higgs and Pudritz, 2007, 2009). By justifying a nonrandomly selected optimal set of amino acids for life, other analytical approaches have suggested the involvement of factors such as “rotational flexibility around the peptide bond” (Koca *et al.,* 1994) and “cost of biosynthetic manufacture” (Cleaves, 2010). For the latter argument, invented amino acids would not have been selected by chance because they require more biosynthetic energy (in ATP) than the simpler meteoritic amino acids (Swire, 2007).

Another proposed universal biosignature uses the relative rates of synthesis for individual chemical species between biotic and abiotic sources. Rates of formation of amino acids synthesized by abiotic processes are constrained by the laws of thermodynamics and kinetics, resulting in a distribution dominated by low-formation-energy, low-molecular-weight molecules. In contrast, the formation of larger amino acids would require the prior formation of larger side chains, which ultimately produce lower yields due to their own kinetic barriers and greater number of isomers. Therefore, unlike biotic (enzymatic) synthesis expending energy to synthesize isomers for amino acids needed for survival and competition, an abiotic synthesis could produce much smaller quantities of many isomers of low-molecular-weight amino acids (Dorn *et al.,* 2011). However, assuming that large-sized amino acids can, in theory, form abiotically (Tyr and His can be formed from CO, ND_3_, and D_2_ by a variant of the Fischer-Tropsch synthesis, and Tyr can also be formed by high-temperature synthesis but not the large Phe and the small Thr [Hayatsu *et al.,* 1971]), if this assumption is true, the proposed size-based amino acid biosignature [identified as a chromatographic pattern of a few dozen peaks, which also comprise the only subset of the possible molecules, referred to by McKay (2004) as the Lego Principle] does not differentiate abiotic from biotic amino acids both in terms of size and Gibbs free energy of synthesis (ΔGr).

Previous analyses on nature's background pool of the 73 amino acid alternatives for life^1^ have also been questioned by applied chemoinformatics and structure-generation studies in relation to the criterion of isomer space surrounding the encoded amino acids, mostly because they reveal far more possibilities than previously imagined (Meringer *et al.,* 2013). Subsequent chemistry analysis of 10^8^ random sets of 20 amino acids from a computational library of 1913 alternative amino acids that lie within the molecular weight range of the encoded amino acids revealed the following: When compared (within their chemistry space coverage) simultaneously in size, charge, and hydrophobicity (a much wider extension of previous approaches) with the standard amino acid alphabet, only six sets with better coverage out of the 10^8^ possibilities were detected. When the six sets were compared with the coded set in terms of their total heats of formation (Δ*H*_f_°), no alternative set was less energetically costly (Δ*H*_f_° more negative). Based on these criteria and noting that no functional criteria (certain functional amino acid groups) were explicitly considered as dimensions of chemistry space in their analysis, it was concluded that the encoded amino acids might represent a largely global optimum, such that any aqueous biochemistry would use a very similar set (Ilardo *et al.,* 2015).

However, most of the current focus on the use of amino acids for extraterrestrial life detection is mainly on the simple amino acids. A good example is the “Signature 17” (17 amino acids) standard (Creamer *et al.,* 2017), which looks for 17 amino acids consisting of 14 (in seven enantiomer pairs) and 3 (achiral) amino acids. The key biomarker aspects of this approach are described below.

### 1.2. Relative concentrations of small meteoritic α-amino acids that are present in Earth life

**FIG. 1.**
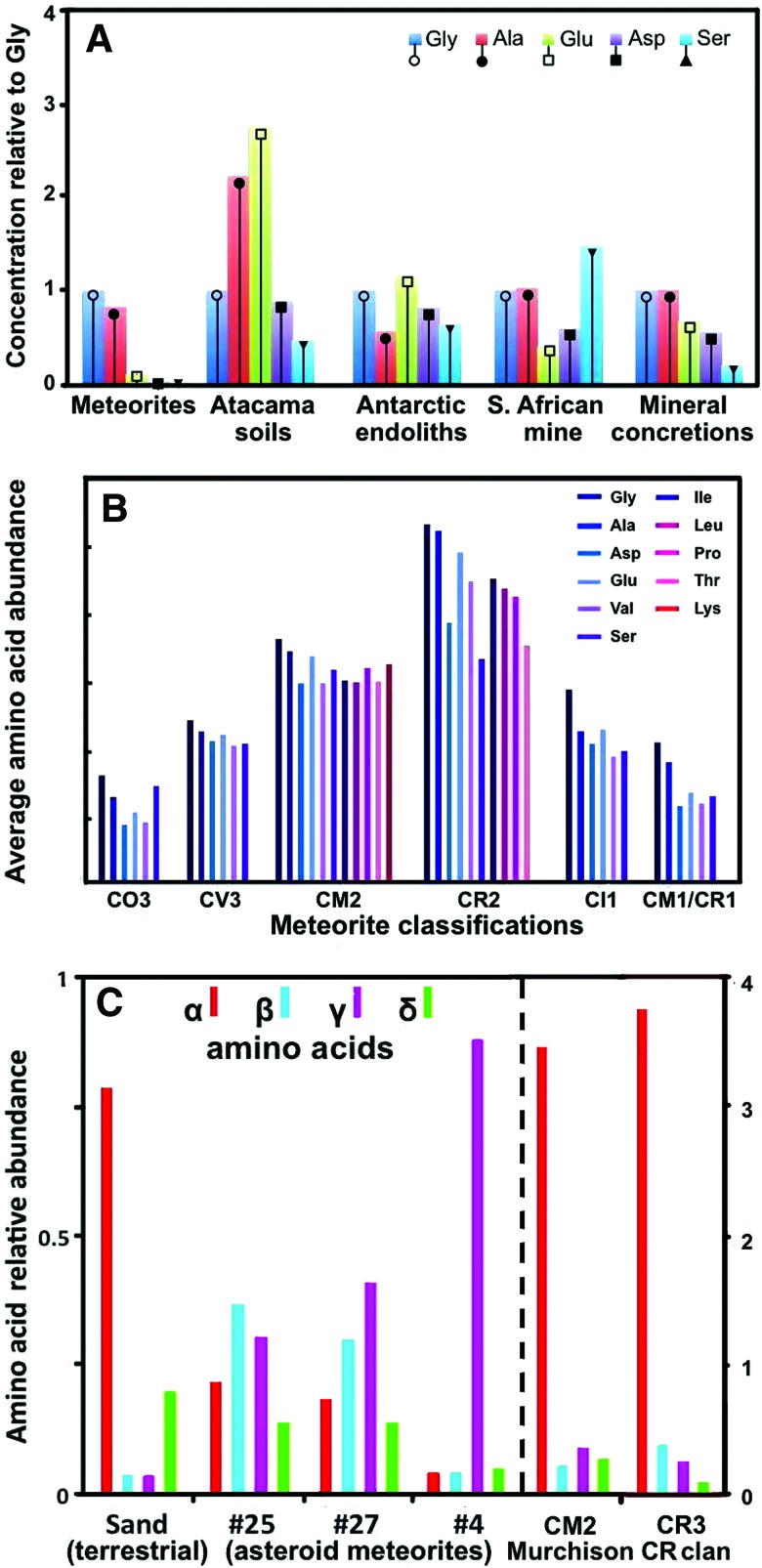
Distribution patterns of amino acids. (**A**) Relative abundances of the simplest meteoritic (abiotic) α-amino acids and in various biogenic samples [drawing modification adapted from elsewhere (Davila and McKay, 2014)]. (**B**) Distribution of 11 meteoritic α-amino acids found in the various meteorite classifications and also in Earth life [drawing modification adapted from elsewhere (Cobb and Pudritz, 2014)]. (**C**) Distribution of C_5_ amino acids by amine position (at α-, β-, γ-, or δ-C atom) in three Almahata Sitta fragments (#4, #25, #27) of asteroid 2008 TC_3_, and in (Sudan) sand. In the latter, biogenic sample, the α-type amino acids prevail, as they also do in the Murchison (CM2) and CR clan (CR3) meteorite abiogenic samples [drawing modified from elsewhere (Burton *et al.,* 2011, 2013, 2015)]. Higher relative abundance of α-amino acids is also seen in other abiogenic sources such as the CR2, CR3, and CH3 carbonaceous chondrites (Burton *et al.,* 2012b, 2013, 2015). Color graphics available at www.liebertonline.com/ast

In terms of universal biomarker applicability for our solar system, all the above approaches converge practically in the search for small amino acid distribution patterns that clearly differ from the abiogenic ones (generated via a Miller-Urey and Fischer-Tropsch type synthesis, or in carbonaceous chondrites). Various approaches have been proposed to search for nonrandom biogenic patterns of such amino acids. One way is the distribution of the relative abundances of the simplest five meteoritic α-amino acids, which are also preferred by Earth life. In an example of this approach (Davila and McKay, 2014), the distribution follows a half-bell-shaped pattern (Gly > Ala > Glu > Asp > Ser; Fig. 1A). However, a less steep but similar pattern is observed in a terrestrial sample (mineral concretions), while the distribution patterns are random in the other tested biogenic samples (Fig. 1A). Moreover, such patterns concern only a small fraction of the (11) abiogenic α-amino acids that also appear in terrestrial life (Cobb and Pudritz, 2014).

Cobb and Pudritz (2014) also noted that Gly predominates in the distribution of the 11 α-amino acids in the various meteorite classifications (CO, CV, CM, CR, CI) (Cobb and Pudritz, 2014), while the patterns of the other amino acids are random (Fig. 1B). Other studies were extended to the distributions of α-, β-, γ-, and δ-amino acids in meteorites in comparison to terrestrial samples (Burton *et al.,* 2012b, 2013, 2014, 2015). Examples are the patterns of the different amino acid types observed in various fragments of the Almahata Sitta meteoritic body of asteroid 2008 TC_3_ (compared to a sand sample from the related strewn field in the Nubian Desert of Sudan) (Burton *et al.,* 2011), and in metal-rich CH and CB carbonaceous chondrites from Antarctica (Burton *et al.,* 2013). The asteroid fragments show lower relative abundance for α-amino acids, and random distribution for the other types, while in the biogenic amino acids isolated from sand the α-type predominates (Fig. 1C). However, higher relative abundance for α-type amino acids is not typical only to biogenic sources because it is also found in the CM2 (Murchison) (Fig. 1C) and the CR2, CR3, and CH3 meteorites (Burton *et al.,* 2012b, 2013).

### 1.3. Amino acid chirality

Homochiral organic molecules (either all D or L) are an important feature of Earth life. In terrestrial biochemistry, only the L-enantiomer of amino acids is used for peptide biosynthesis. Therefore, a significant left or right imbalance, defined as (R − L) × 100%/(R + L), has been proposed to be a potential biosignature for the search for extraterrestrial life, in light of the fact that chemical synthesis of amino acids produces a racemic mixture (D/L 50/50). In terrestrial fossil life, D/L values of different amino acids can be arranged in an order according to their racemization rates. In abiotic synthesis, enantiomeric excess is due to aqueous processing and usually does not follow the racemization rates. Another important aspect of amino acid chirality is that the enzymes are also chiral, because they must be extremely stereoselective to produce or bind in their active site only one of the two enantiomeric reactant forms. In bacteria, for example, D-amino acids are even used as a source of nitrogen by running enzymatic racemization in reverse when soils are inundated with racemic amino acids. Here, resident bacteria consume both D- and L-enantiomers (simultaneously or sequentially depending on the level of their racemase activity), thus maintaining terrestrial environments very low in D-amino acids except for those produced by racemization (Zhang and Sun, 2014). Moreover, D-amino acids are present in all kingdoms of terrestrial life and are involved in diverse physiological functions (Martínez-Rodríguez *et al.,* 2010). Amino acid chiral excess distribution, therefore, becomes an uncertain criterion if applied alone for searching for extinct life, because the enantiomeric unequal distribution of organics is not a unique biogenic process even for Earth life.

Chirality origins have been associated with chance, local chiral influences, parity violation in weak interactions, the salt-induced peptide formation (SIPF) reaction, and stereoselectivity of the SIPF reaction (Fitz *et al.,* 2007). Indeed, abiogenic chirality is not an uncommon phenomenon. Polarized UV radiation and catalysis on the surface of specific crystals are known to produce up to 1% amino acid chirality (Modica *et al.,* 2014), which, although initially tiny, may be amplified by prebiotic mechanisms to explain the high chirality (exceeding 60%) observed for certain amino acids in carbonaceous chondrites (Glavin and Dworkin, 2009; Glavin *et al.,* 2012; Tarasevych *et al.,* 2015). Neutrino-induced selective ^14^N nuclear destruction in D-enantiomer amino acids would produce molecular chiral selectivity via modification of the magnetic field at the nucleus by electric fields, creating nuclear magnetizations that are asymmetric in chirality. The selective destruction of D-enantiomers could result in chiral excesses sufficient to drive subsequent autocatalysis which can produce the few-percent enantiomeric excesses and subsequent homochirality in meteorites (Famiano *et al.,* 2018).

The first study that challenged the view that chiral compounds of meteorites occur as racemic mixtures was the detection of enantiomeric excess (L-) in four amino acids typically found in the Murchison meteorite (Cronin and Pizzarello, 1999). Enantiomeric excess (L-) was also observed in other Murchison indigenous amino acids by another study of the same group (Cronin and Pizzarello, 1997). Significant enantiomeric enrichments for some L-amino acids (*e.g.,* Asp and Glu) have been found in meteoritic fragments that fell on the frozen Tagish Lake in Canada (Glavin *et al.,* 2012). Some meteorites contain amino acids that share, to some degree, the trait of chiral asymmetry with biomolecules. These are the α-methyl-α-amino acids that display L-enantiomeric excesses (Pizzarello *et al.,* 2006). Nonracemic isovaline was observed in carbonaceous chondrites exposed to aqueous alteration (Murchison, Orgueil, and SCO 06043), consistent with the theory that liquid water played an important role in amplifying small L-isovaline excesses on the parent bodies (Glavin *et al.,* 2011). Moreover, it has been shown that liquid water, present in Mars (*e.g.,* as nanometer-thick layers on mineral grains), will greatly accelerate the racemization of amino acids if they have been produced by an extinct martian biota at an enantiomeric excess (Bada and McDonald, 1995).

The work of Glavin *et al.* (2012) on the Tagish Lake provides probably the best and most contamination-free results, showing that amino acid chiral excess may not be a reliable life biomarker, as it can easily arise from the already mentioned variety of physical or chemical processes. What has been proposed as a more reliable life biomarker is homochirality, that is, the common chirality of a proteinogenic set of amino acids of Earth life. Ideally, it is the homochirality of the 19 amino acids used in Earth life (excluding achiral amino acids Gly, β-Ala, and GABA). Most approaches focus on five (D/L-Ser, -Val, -Ala, -Glu, and -Asp) to seven (D/L-Ala, -Asp, -Glu, -His, -Leu, -Ser, -Val) enantiomer pairs (Creamer *et al.,* 2017). This seems to be the case with the ESA/Roscosmos ExoMars mission, although it is not clear what they mean by enantiomeric excess (chirality) (Goesmann *et al.,* 2017). Nonetheless, for this mission the discovery of >20% amino acid enantiomeric excess will require further corroborating evidence from other biomarkers. These can be lipids, also to be analyzed by the mission's MOMA instrument, as long as they are identified as biomarkers under the minimum hydrocarbon chain length criterion previously proposed (Georgiou and Deamer, 2014). However, even homochirality has been strongly criticized as an extraterrestrial life-detection criterion, on the basis that it is needed only for systems that are directly connected to evolutionary requirements for encoding or precise folding. This is not true for non-encoded biopolymers to fold or function, given that the natural antibiotic gramicidin, a 15-amino-acid biopolymer, has approximately half D-amino acids and yet still folds precisely into an ion-conducting channel (Benner, 2017).

## 2. Functional Properties of Amino Acids: A New Approach

According to the aforementioned approaches in establishing universal criteria for life, synthesis least energy cost and best chemistry space fit favor the prevalence of the coded set of simple amino acid distributions (by comparison to meteoritic ones) for Earth and possibly extraterrestrial life in surviving nature's selection forces. These criteria are based on thermodynamic properties that rely mostly on the structural characteristics of individual amino acids, but they are not conclusive to differentiate their abiogenic or biogenic origin. As useful as these criteria may be for supporting the search for amino acids in our solar system, they need to be corroborated with universal characteristics more exclusive for life.

Organic catalysis (constrained in space and volume for Earth life) can be such a universal characteristic. Life cannot be envisioned without organic catalysis because biochemical reactions can take an extremely long time to complete if noncatalyzed. Earth life has bypassed this problem by devising organic catalysts (protein enzymes) to accelerate its biochemical reactions. In general, spontaneous (noncatalyzed) rate constants of organic substrates in Earth life can be accelerated by organic catalysts from 10^6^- to 10^17^-fold (Horton *et al.,* 1993; Radzicka and Wolfenden, 1995). Thus, organic catalysis must be a universal feature of water-based extraterrestrial life as well.

Therefore, biogenic differentiation of amino acids from abiogenic can be searched in the cumulative functional (catalytic and/or structural) advantages they and certain groups in their side chains can offer to terrestrial and by extension to extraterrestrial life. This functional approach has also been applied for lipids as biomarkers for extraterrestrial life (Georgiou and Deamer, 2014). Some general structural considerations on Earth and cometary α-amino acids are a useful introduction for this approach.

### 2.1. Structural considerations on α-amino acids

Earth life makes/uses only α-amino acids for protein synthesis and small amounts of β*-* (*e.g.,* β-aminoisobutyric acid) and γ- (*e.g.,* γ-aminobutyric acid) amino acids for other functions. Abiogenic chemistry, on the other hand, produces a mixture of all three. Earth life protein amino acids must be of the α-type, as they can form easily rotating, hydrogen bond–generating horizontal planes of α-imino (N-H)-α-carbonyl (C=O) peptide bond polymers by linear translation systems (ribosomes). Beta-peptide bonds can be made post-translationally for peptide crossing or branching modifications. However, instead of the 11 (also abiogenic) α-amino acids that Earth life has adopted to synthesize the peptide bonds in proteins (Cobb and Pudritz, 2014), meteoritic amino acids could have provided primitive life with at least 30 α(or 2)-amino acid alternatives [Table 1; compiled from Burton *et al.* (2012b)]. Was, then, the selection or adoption by Earth life of only 11 meteoritic α-amino acids random?

**Table 1.** Meteoritic α(2)-Amino Acid Alternatives to Earth Life's Amino Acids

*Hydrophobic straight side chains of C2 to C7 length*	*Branched hydrophobic chains of ≥5 carbon atoms*
norvaline	2-amino-3-ethylpentanoic acid
norleucine	2-amino-3,3-dimethylpentanoic acid
2-aminoheptanoic acid	2-amino-3,4-dimethylpentanoic acid
α-aminobutyric acid	2-amino-4,4-dimethylpentanoic acid
*Two hydrophobic side chains*	2-amino-3-methylhexanoic acid
α-aminoisobutyric acid	2-amino-4-methylhexanoic acid
2-amino-2-ethylbutanoic acid	2-amino-5-methylhexanoic acid
2-amino-2,3-dimethylbutanoic acid	*Alternatives to Glu and Asp*
2-amino-2,3,3-trimethylbutanoic acid	α-aminoadipic acid
2-amino-2-ethyl-3-methylbutanoic acid	α-aminopimelic acid
2-amino-2-ethylpentanoic acid	2-methylglutamic acid
2-amino-2,3-dimethylpentanoic acid	3-methylaspartic acid
2-amino-2,4-dimethylpentanoic acid	*Alternatives to Lys*
2-amino-2-methylhexanoic acid	2,4-diaminobutanoic acid
cycloleucine	2,3-diaminobutanoic acid

**FIG. 2.**
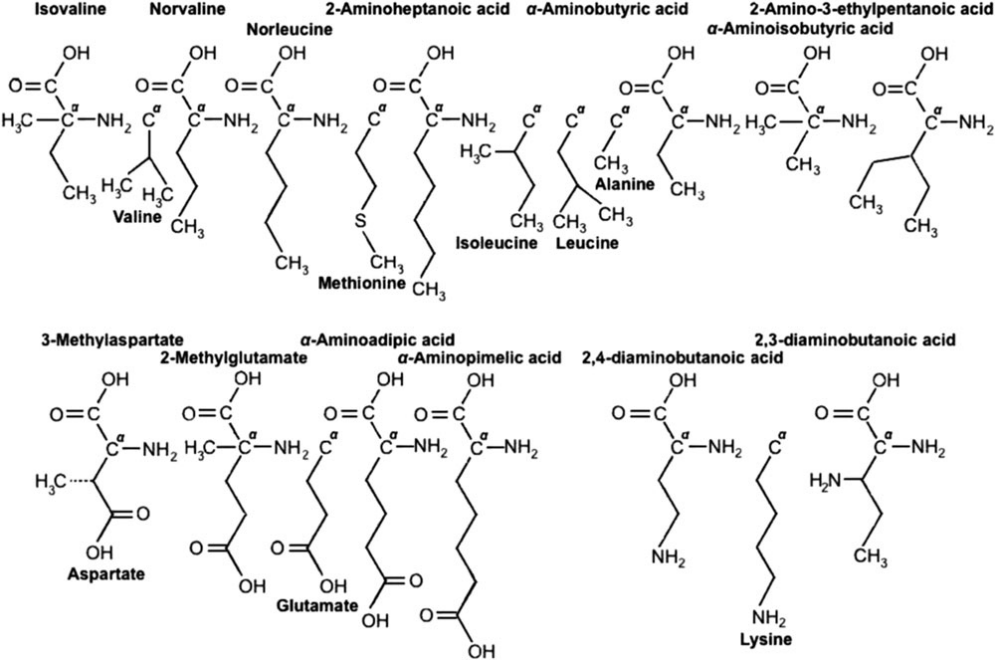
Side chains of meteoritic α-amino acids not found in proteins, juxtaposed with those of protein amino acids that they could have replaced in an alternative amino acid set.

Meteorites contain a variety of hydrophobic α-amino acids, having straight side chains with lengths ranging from 2 to 7 carbon atoms (including the α-carbon), two side chains attached at the α-carbon, and even branched hydrophobic chains with ≥5 carbon atoms (after the α-carbon) (Table 1). All these amino acids could have served as alternatives to the hydrophobic Ala, Val, Ile, and Leu (the non-meteoritic Met, Phe, Trp, and Tyr included) (Fig. 2). Their non-appearance in Earth life could suggest that amino acids having branched hydrophobic chains with a maximum number of 4 carbons or single-carbon side chains are the preferred ones. That is, 2- to 4-carbon branched nonpolar chains (Val, Ile, Leu) are preferred over those with 3-, 4-, 5-carbon straight chains (norvaline, norleucine, 2-aminoheptanoic acid). Branched side chains with higher than 4 carbons would hinder α-helical conformations. This has been shown in studies on the stability of the α-helix in water by straight-chain and branched nonpolar amino acids (Padmanabhan and Baldwin, 1991). Another reason is that amino acid short branched hydrophobic chains, as with short branched lipids (*e.g.,* in archaebacteria), are more fluid at low temperatures, promoting, thus, the conformational flexibility of proteins.

Earth life invented or selected, instead, another kind of large side chains, the three versions of aromatic groups (Phe, Trp, and Ty), apparently to provide flexible and strong hydrophobic interactions not for α-helicity but for optimal positioning of substrates in the catalytic sites of enzymes. Moreover, Earth life excluded amino acids with more than one side chain attached to the α-carbon (by keeping hydrogen as the fourth substituent group in the α-carbon, even in Pro), as they hinder α-helical formation. As in the case with lipid membrane hydrocarbons (Georgiou and Deamer, 2014), hydrophobic small branching ensures greater freedom in folding for protein structure stability, especially at extreme conditions (*e.g.,* low temperatures). On the other hand, the meteoritic amino acid alternatives to Glu and Asp (Table 1, Fig. 2) were not selected by Earth life possibly because certain structural features in their side chains compromise the catalytic functionality of their terminal carboxyl groups: These are (i) the existence of the hydrophobic methyl group in the β-C atom of 3-methylaspartate (where the carboxyl group is attached) or in the α-C atom of 2-methylaspartate and (ii) the quite distant position of the carboxyl group in the δ-C atom of α-aminoadipic or in the ɛ-C atom of α-aminopimelic acid (in contrast to the γ-C atom of Glu). Finally, although 2,4-diaminobutanoic and 2,3-diaminobutanoic acid could have served as alternatives to Lys (Table 1, Fig. 2), Earth life had to incorporate a unique amino acid with a terminal amino group at the ɛ-C atom [Lys was found only in the CM2 meteorite (Cobb and Pudritz, 2014)]. There are at least two reasons for this: Lys ɛ-NH_2_ group (i) can acquire pKa values (5.3–10.4) comparable to those of its naturally buried (in proteins) ionizable groups that are involved in catalysis and biological H^+^ transport (energy transduction) (Isom *et al.,* 2011) and (ii) this group has higher proton affinity than the γ-NH_2_ group of Lys's meteoritic counterpart 2,4-diaminobutanoic acid (Schroeder *et al.,* 2004). Since Earth life selected or adopted almost a third of the available abiotic α-amino acids, leaving the majority behind during the course of early evolution, it could be hypothesized that they were not selected by chance and this should hold true for the invented amino acids.

### 2.2. Distinguishing abiogenic from biogenic amino acids

Assuming that the α-type of amino acids is a universal feature for peptide bonds, we need to extend our approach to amino acid distribution patterns based on biochemically universal functional properties for life, terrestrial and extraterrestrial. Such properties can be found in the amino acid side-chain structures, the types of their terminal groups and their positioning in the side chain, and also on the degree their modifications regulate flexibility in protein folding and activity. In other words, the universality of such terminal groups for life exists if they can maintain their functionality even when integrated in other types of side-chains than those found in Earth-life amino acids, and even in non-amino-acid organics. Such “new” amino acids could, then, be part of other sets suitable for any kind of extraterrestrial life. After all, even the set of the 20 amino acids is not fixed for Earth life, as it is possible to expand experimentally the natural set of amino acids by expanding the eukaryotic genetic code (Chin *et al.,* 2003). This has resulted in incorporation into proteins of non-natural amino acids such as *p*-nitrophenylalanine, *o*-methyl-tyrosine, *m*-iodotyrosine 2-naphthylalanine, *p*-azidophenylalanine, and *p*-benzoylphenylalanine, with others such as *o*-(trimethylammoniumalkyl) tyrosine that extend protein function (Hohsaka and Masahiko, 2002). Another way Earth life creates unusual functional amino acids is by post-translational modifications of the existing ones such as phosphorylation. Moreover, functional terminal groups need not be harbored only in amino acids but also in other organic molecules like ribozymes that function as catalysts.

Are there any functional features in the abiogenic (meteoritic) and invented amino acids that can be universal for life? The present study proposes the following functional propensities: for (1) biochemical catalysis, (2) intramembrane α*-*helicity, and (3) protein adaptations to extreme conditions (thermophilic, psychrophilic, halophilic).

### 2.3. Organic catalytic potential: Amino acid terminal catalytic groups

In Earth life, water allows two opposite catalytic processes to take place: dehydration synthesis and hydrolysis to either build or break down biological molecules. These processes must be universal for any water-based life in the Universe. Most biopolymers of Earth life are made by dehydration synthesis, by which two biomolecules are catalytically bonded (condensed) by a loss of one molecule of water. In hydrolysis (*e.g.,* of esters and amides), the bond between monomers or within an organic molecule is catalytically broken by the addition of one molecule of water, with an O-H bond in the water molecule also being broken. Then, the -OH group from the water molecule adds to one part from the organic molecule, and the H atom to the other.

For Earth life to be established, dehydration synthesis and hydrolysis constitute key universal anabolic and catabolic catalytic processes, respectively. However, reactant concentration is a crucial parameter for organic catalysis, thus for prebiotic chemistry as well. Since water is ever-present as a competing nucleophile, high reactant concentrations are often needed to favor product formation over hydrolysis. Water-based extraterrestrial life in its primitive stage may have adapted to existing low reactant concentrations in extreme environments and, consequently, to a biochemistry with higher tendency for hydrolysis.

The present study proposes to use hydrolytic potential to estimate the probability that an organic sample contains biomarkers for extraterrestrial life. This can be done by identifying certain catalytic groups in amino acids and other organic compounds, as described below.

#### 2.3.1. Hydrolytic potential

Hydrolytic potential of extraterrestrial organics can be estimated from assays that test for selected certain catalytic activities. The catalytic groups can be identified in extraterrestrial organic samples by methods described elsewhere (Badalassi *et al.,* 2000; Wahler *et al.,* 2001; Albada and Liskamp, 2008), and these can be easily adopted for spacecraft instrumentation. For instance, organic samples can be tested for catalytic activity related to hydrolysis by performing assays with fluorescent substrates such as umbelliferone or 6-methoxynaphthaldehyde for ultrasensitive detection and quantification. Although this approach is not based on enzymatic catalysis, the catalytic groups of extraterrestrial life are likely to be similar to the side-chain catalytic groups of certain amino acids in the active centers of enzymes. These amino acids can also be explored as additional universal biomarkers for extraterrestrial life.

**FIG. 3.**
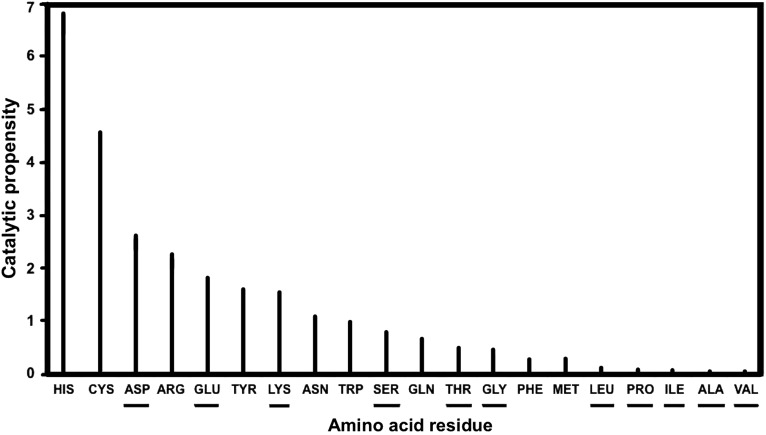
Graphical illustration of the catalytic propensity of Earth life amino acids versus those also produced abiogenically. Catalytic propensity is defined as the ratio of the percentage of catalytic residues constituted by a particular residue type, over the percentage of all residues constituted by the same particular residue type (Bartlett *et al.,* 2002). Underlined are the amino acids found in meteorites and made by simulated hydrothermal prebiotic chemistry. The drawing is a modification adapted from elsewhere (Bartlett *et al.,* 2002).

Amino acids bestow catalytic activity to enzyme active centers by certain terminal groups positioned at the end of their side chains. Most important is the catalytic role of their terminal groups that function either alone or as catalytic triads in the enzyme active centers. Amino acids adhere to the following criteria for classifying them as catalytic active site residues: (i) direct amino acid involvement in the catalytic mechanism (*e.g.,* as a nucleophile); (ii) exerting an effect on another residue or water molecule that is directly involved in the catalytic mechanism (*e.g.,* by electrostatic or acid-base action); (iii) stabilization of a transition-state intermediate; (iv) exerting an effect (steric and electrostatic included) on a substrate or cofactor that aids catalysis (*e.g.,* by polarizing a bond to be broken) (Bartlett *et al.,* 2002). From a database of 178 enzymes, it has been found that 65% of the catalytic residues are from ionic groups (His, Arg, Lys, Glu, Asp), 27% from polar groups (Gln, Thr, Ser, Asn, Cys, Tyr, Trp), and only 8% from the hydrophobic group of the amino acids (Bartlett *et al.,* 2002) (Fig. 3). A similar study on a set of 191 enzymes identified 11 polar and charged amino acids engaged in catalysis. These carry the chemical groups imidazole (His), guanidinium (Arg), amine (Lys), carboxylate (Glu, Asp), amide (Gln, Asn), hydroxyl (Ser, Thr, Tyr), and thiol (Cys). This is an expected conclusion since catalysis involves the movement of protons, electrons, and charge stabilization by electrostatic forces provided by charged and/or polar residues (Gutteridge and Thornton, 2006). In terms of hydrolytic catalysis, the protonated forms of His, Asp, Glu, Tyr, Cys, and Lys can function as acids in acid-catalyzed hydrolysis, which involves a partial proton transfer from a proton donor to a substrate. Base-catalyzed hydrolysis, on the other hand, can be performed by the unprotonated forms of Asp, Glu, His, Tyr, Cys, and Lys.

The following conclusions can be drawn: His and Cys (both absent in meteorites) exhibit the highest catalytic propensity of all proteinogenic amino acids. They constitute 18% and 5.6% of all catalytic residues but are only 2.7% and 1.2% of amino acids in proteins, respectively. Their catalytic propensities are 6.7 (18/2.7) and 4.7 (5.6/1.2) and are the highest (Fig. 3). The ranking of His is expected, as this amino acid can be both neutral or positively charged at physiological pH 7 and plays an active role in hydrolytic reactions by acting as nucleophile, or engaged in acid-base hydrolysis, for example, of ester bonds. His and Cys have the closest pKa values to intracellular pH, and this may explain their high degree of catalytic potential (Bartlett *et al.,* 2002). Next in the ranking are the charged amino acids Arg, Asp, Glu, and Lys (underlining designates presence also in meteorites). The pKa values of Asp and Glu carboxyl groups provide charges which affect other amino acid residues and the substrate. On the other hand, the three nitrogen groups in the side chain of Arg can be involved in more electrostatic interactions than just the one group of Lys. Thus, they can be positioned more accurately to facilitate catalysis; and, because of their favorable geometry, they stabilize a pair of oxygen atoms on a phosphate group, which is a common catalytic interaction in metabolism of Earth life (Bartlett *et al.,* 2002). The polar Ser, Thr, Tyr, Gln, and Asn are less often used as catalysts (Gutteridge and Thornton, 2006).

Cys is of special interest because, despite its ionization to the thiolate anion, it is uncommon on the surface of proteins and it is typically buried inside proteins. Sulfur, unlike oxygen, has a low propensity to form hydrogen bonds, and the Cys thiol group when oxidized forms disulfide bonds with other thiol groups. Sulfur-containing Met has the lowest catalytic propensity among the invented amino acids probably because of its nonpolarity and high hydrophobicity, which restrict this amino acid in the interior of proteins.

Most important in enzymatic active sites is not the catalytic propensity of their constituent active groups but the combination of two or more residues to form *catalytic units*. These units serve similar roles in catalytically unrelated enzymes. Most frequent are the catalytic triads *Ser-His-*Asp (in, *e.g.,* hydrolase serine protease), *Thr-His-His* (*e.g.,* hydrolase phosphotransferase), *Ser-His-Glu* (combined with Glu-Glu, *e.g.,* in hydrolase cellulase), *Asp-Tyr*-*Lys* (*e.g.,* aldo-keto reductase), *Thr-Lys-Asp* (*e.g.,* the hydrolase L-asparaginase), *Lys-Glu-Lys* (*e.g.,* indole-3-glycerol-phosphate synthase) (Gutteridge and Thornton, 2006). Of special interest are the catalytic units based on the highest in catalytic propensity imidazole group of His. The reason is that (i) they are widely shared by many hydrolytic enzymes (*e.g.,* serine hydrolase, lipase, esterase, asparaginase, β*-*lactamase) (Dodson and Wlodawer, 1998; Bornscheuer and Kazlauskas, 1999), (ii) they exist in triad variations (*e.g., Ser-His-Asp, Ser-His-Glu,* *Thr-His-His*) that reflect their wide catalytic spectrum (Ekici *et al.,* 2008), and (iii) they reveal a catalytic universality in the way chemical constraints can independently select for the same catalytic solutions (Buller and Townsend, 2013). The second-highest in catalytic propensity is Cys, which can also participate in hydrolysis-involved dyad catalytic units with Ser or Thr (*Cys-Ser, Cys-Thr*). This is true for all phosphagen kinases (Lyzlova and Stefanov, 1990; Wang *et al.,* 2001), which catalyze the reversible transfer of a phosphate between ATP and guanidino substrates (*e.g.,* creatinine)—a reaction that is central to cellular energy homeostasis (Azzi *et al.,* 2004). It should be noted that the meteoritic amino acids with the highest propensity (Asp, Glu, Lys, Ser, and Thr) can also join forces themselves as catalytic triads (*Thr-Lys-Asp, Lys-Glu-Lys*), besides participating in the catalytic units (triads or dyads) of the invented His, Cys, and Tyr.

Not all amino acids possess such terminal catalytic groups, especially the majority of the meteoritic ones. For instance, the small meteoritic amino acids Gly, Ala, Val, Leu, Ile, and Pro do not meet the complex catalytic needs of Earth and possibly of extraterrestrial life, when judged by their very low catalytic propensities or their nonparticipation in catalytic units either among themselves or in combination with high catalytic propensity invented amino acids. Nonetheless, some of the meteoritic amino acids could have served as primitive catalysts by acting either as single entities [*e.g.,* Ala and isoVal, present in some meteorites (Pizzarello and Weber, 2004)] or as primitive di- and tripeptides [*e.g.,* based on Ala, Val, Ser (Zou *et al.,* 2005)] that mediate simple organic reactions such as the direct asymmetric intermolecular aldol reaction (Zou *et al.,* 2005). Thereafter, Earth life had to invent more versatile catalytic groups to meet its more complex biochemical requirements. These are mainly the imidazole terminal group of His and the thiol terminal group of Cys, complemented by the terminal groups guanidinium of Arg and amide of Gln and Asn. The catalytic maximization of these groups is facilitated by the abiogenic amine (Lys), carboxylate (Glu, Asp), and hydroxyl (Thr) groups.

**FIG. 4.**
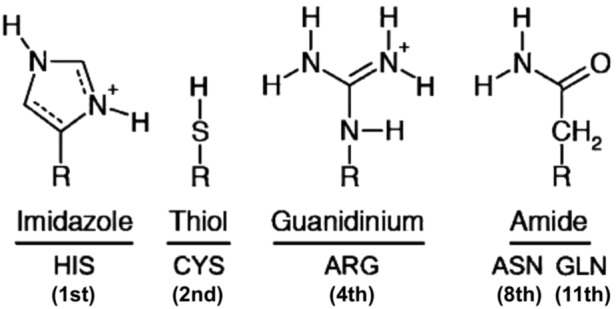
Terminal catalytic groups in side chains of amino acids exclusively found in Earth life. Numbers in parenthesis indicate their ranking order in catalytic propensity (shown in Fig. 3).

*Therefore, the amino acid groups imidazole, thiol, guanidinium, and amide can serve as unique universal biomarkers because they are not found in the meteoritic amino acids (Fig. 4). They could also serve the catalytic needs of extraterrestrial life either as terminal groups in amino acids, or in a different set of amino acids carrying these same catalytic groups in side chains that may differ in length, branching, substituent or pendant groups. However, these same catalytic groups need not be carried by protein-forming amino acids, as they could be part of other organic complexes or even small abiogenic peptides*.

Besides its widespread participation in the catalytic triads of many hydrolytic enzymes, the catalytic universality of imidazole is also illustrated by its wide use in organic compounds that are catalysts. These consist of carbon, hydrogen, sulfur, and other nonmetal elements, often based on cyclodextrin (Breslow, 2006; Bogliotti and Dalko, 2007) or not (Jarvo and Miller, 2002; Albada and Liskamp, 2008). These catalytic alternatives illustrate the range of organic catalysts that could participate in primitive life biochemistry (Zhang *et al.,* 2010).

In a second extraterrestrial amino acid set, Asp and Glu could have been replaced by their meteoritic counterpart amino acids α-aminoadipic and α-aminopimelic (Fig. 2). However, since Asp and Glu are found both in meteorites and Earth life, they are not exclusive biomarkers, nor is their terminal carboxylate group. Similarly, the terminal hydroxyl group is not an exclusive biomarker, as it is carried by the meteoritic/biogenic Ser. Lys (ɛ-NH_2_) is another abiogenic amino acid that could be replaced by the meteoritic amino acid 2,4-diaminobutanoic acid (Fig. 2). His, on the other hand, has the highest catalytic propensity and participation frequency in catalytic triads, and, in this respect, can be considered a universal biomarker of life. Similarly, any unknown amino acid that carries imidazole as its terminal group should be considered, given the fact that such amino acids (His included) have not been identified in meteorites. Other exclusive functional terminal groups are the thiol (in Cys), guanidinium (in Arg), and amide (in Gln and Asn) (Fig. 4). Therefore, extraterrestrial life can rely on different sets of amino acids as long as they carry some or all of the aforementioned exclusive terminal catalytic groups. It is not the structure of the carrier side chains that needs to be preserved in these different sets, but only their catalytic terminal groups.

*It is proposed to look for those Earth-like amino acid distributions that have the highest catalytic propensity (Fig. 3), with emphasis on His because it carries the catalytic terminal group imidazole. These distributions also include amino acids that carry the catalytic terminal groups thiol (Cys), guanidinium (Arg), and amide (Asn, Gln). A complementary proposition is to look at frequency distributions among the amino acids participating in the catalytic triads involving His (Ser-His-Glu and Ser-His-Asp, and Thr-His-His) and Tyr (Asp-Tyr*-*Lys*)*, and dyads involving Cys (Cys-Ser*, *Cys-Thr). These can serve as complementary distributions of the abiogenic amino acids Thr, Lys, Asp, and Glu, because they also form catalytic triads (Thr-Lys-Asp and Lys-Glu-Lys). An expected biomarker pattern would involve similar concentrations within the distributions of such amino acids.*

The ExoMars 2020 Organic Molecule Analyser (MOMA) will have the capability to identify both invented and abiotic amino acids in samples acquired from depths of up to 2 m below the martian surface (Goesmann *et al.,* 2017). Nearby or isolated sulfide minerals and iron-magnesium silicates upon dissolution may contain enzymatic cofactors like Cr, Cu, Fe, Mg, Mo, Ni, and Zn (Banfield *et al.,* 1999; Rogers and Bennett, 2004; Röling *et al.,* 2015). *High localized concentrations of metals with catalytic activity could also serve as complementary biomarkers.*

Comparing the respective stable sulfur isotope values of biogenic Cys and Met to those of sulfate-containing minerals of similar ages could provide an alternative approach for determining the occurrence of past extraterrestrial life (Engel, 2007).

### 2.4. Amino acid propensity for oligopeptide intramembrane α-helicity

Enzymatic catalysis takes place both in the cytoplasm and in cell membranes by embedded proteins. The secondary and tertiary structures of enzymes use many peptide configurations (α-helices, β-sheets, helix turns) especially when present in aqueous or charged environments such as α-helical segments in cytoplasmic or ribosomal proteins, respectively. This is due to the fact that the propensities of the invented amino acids in forming α*-*helices are not coupled to their hydrophobicity. In contrast, protein amino acids that are also present in meteorites are unique in that they have high propensity for α-helicity coupled to their hydrophobicity, especially in intramembrane environments. The most hydrophobic of the meteoritic amino acids have a high α*-*helicity propensity for transmembrane segmentation, which is uncoupled from their hydrophobicity when compared with their α-helicity propensity in an aqueous environment (Liu and Deber, 1998). Based on the calculated rank of α-helicity propensities [Table 2, composed from previously published data (Liu and Deber, 1998)], the meteoritic amino acids Ile, Leu, Val, and Ala, and the large invented amino acids Phe, Met, Tyr are ideal in making α*-*helices for membrane proteins. Leu, Val, and Ala have been found to form dipeptides (*e.g.,* Ala with itself or with Asp, and Val with Gly) in carbonaceous chondrites of the CM2 type (Shimoyama and Ogasawara, 2002). *Since oligopeptides, for example, of α-helix-length have not been found in meteorites, this can be a universal feature for proteinogenic amino acid–based extraterrestrial life*.

**Table 2.** Amino Acid Propensity for α*-*Helicity

	*Meteoritic amino acids*		*Invented amino acids*	
*Rank order*	*Intra-membrane medium*^a^	*Aqueous medium*^b^	*Intra-membrane medium*^a^	*Aqueous medium*^b^
1	**Ile**	Glu		
2	**Leu**	**Ala**		
3	**Val**	**Leu**		
4		**Ile**	**Phe**	
5	**Ala**	Asp		
6			**Met**	Arg
7	**Gly**			**Met**
8			**Tyr**	Gln
9			Cys	**Tyr**
10	Thr			**Trp**
11			**Trp**	**Phe**
12	Ser			
13		**Val**	His	
14		Lys	Gln	Cys
15		Thr	Arg	
16			Asn	His
17	Asp	Ser		
18	Lys			Asn
19	Glu	**Gly**		
20	Pro	Pro		

Table uses ranking data from elsewhere (Liu and Deber, 1998). Propensity rank order numbers (1–20) refer to the set of 20 Earth life amino acids. Hydrophobic amino acids are shown in bold.

^a^ Ranking affected by hydrophobicity.

^b^ Ranking not affected by (uncoupled from) hydrophobicity.

Some of the meteoritic amino acids (*e.g.,* Ala and Val) can play a role of primitive organocatalysts. For example, enantiomerically enriched L-Ala and L-isoVal found in some meteorites were shown to asymmetrically catalyze an aldol condensation of glycolaldehyde in water to give tetroses, with chiral configurations influenced by the chirality of the amino acid catalyst (Pizzarello and Weber, 2004).

*It is proposed that the high propensity for (intra-membrane) α-helicity small abiogenic (Ile, Leu, Val, Ala) and large invented (Phe, Met, Tyr, Trp) amino acids present either as group patterns or in oligopeptides can serve as universal biomarkers for extraterrestrial life*. *For Tyr, in addition, its coexistence also with Asp and Lys may be an indicator of catalytic activity, because, as already stated, these three amino acids can form the catalytic unit Asp-Tyr-Lys in Earth life.*

It should be noted that the invented large aromatic amino acids Phe, Tyr, and Trp exhibit an intrinsic fluorescence at ex/em 260/282, 275/304, 280/353 nm, respectively. *It has been proposed that these amino acids can be detected on the martian topsoil by a modification of the Curiosity rover Mars Science Laboratory ChemCam instrument, which could measure their fluorescence through an onboard spectrometer after UV-laser pulse excitation (Smith* et al., *2014). This technology could be also applied on the plumes of Enceladus and Europa as the spacecraft flies by*.

### 2.5. Implications for the origin of life from intra-membrane α-helicity

It is known that the lipid bilayer component of biological membranes regulates the distribution, organization, and function of bilayer-spanning proteins by interactions between specific lipids and embedded peptide strands, as well as more general interactions between the proteins and the hydrophobic bilayer domain. These modulate the energetics and kinetics of protein conformational transitions, as well as the protein distribution between different membranous compartments. Such regulation arises from the hydrophobic coupling between protein hydrophobic domains and the bilayer hydrophobic core, which causes protein conformational changes that involve the protein/bilayer boundary to perturb the adjacent bilayer. Such perturbations incur an energetic cost, which for a given conformational change varies as a function of the bilayer material properties such as thickness, intrinsic lipid curvature, and the elastic compression and bending moduli. As a result, lipid bilayers act as an allosteric regulator of membrane function (Andersen and Koeppe, 2007).

The hydrophobic thickness of the lipid hydrocarbon bilayer is ideally the length transversed by a transmembrane α-helix domain of an embedded protein. If the hydrophobic thickness of the lipid hydrocarbon bilayer is smaller than the hydrophobic length of the α-helix, this hydrophobic mismatching is not thermodynamically favorable because of the energetically unfavorable exposure of hydrophobic amino acid residues to water (Andersen and Koeppe, 2007). A typical hydrocarbon bilayer thickness is ∼30 Å (excluding the ∼10 Å–long glycerol region from the phosphate to the first methylene of the hydrocarbon chain), which corresponds to acyl chain lengths of 16–22 carbons. Given that the length of an α-helix increases by 1.5 Å per amino acid residue (Sharpe *et al.,* 2010), the lipid bilayer length energetically favors α-helices of ∼20 amino acids.

Thus, it is possible that in the first stages of primitive protocell formation these abiogenic amino acids would generate mostly hydrophobic-hydrophilic combinations of small peptides. The primitive lipid bilayer would spontaneously select and incorporate the most hydrophobic α*-*helical oligopeptides, possibly with hydrophilic/polar segments made of Ser, Asp, Glu extending at both ends, which can spontaneously insert themselves across lipid bilayers (Brambillasca *et al.,* 2006). Such transmembrane peptides will stabilize the primitive membranes as long as the length of their α*-*helices matches the thickness of the lipid bilayers (Andersen and Koeppe, 2007), which will be initially formed by the available abiotic short polar hydrocarbons.

Such oligopeptides are known to be made from meteoritic amino acids under conditions where the activity of water has been reduced in association with elevated temperatures. These conditions initiate condensation reactions that lead to peptide formation (Rodriguez-Garcia *et al.,* 2015). Indeed, it has been repeatedly shown in hydrothermal experiments that dipeptides of meteoritic amino acids can form rapidly, as well as small concentrations of tripeptides and longer oligomers. Starting with larger oligomers (*e.g.,* three or four amino acids in length), then these peptides can be further lengthened by hydrothermal reactions with the involvement of the monomers (Pizzarello and Shock, 2010).

**FIG. 5.**
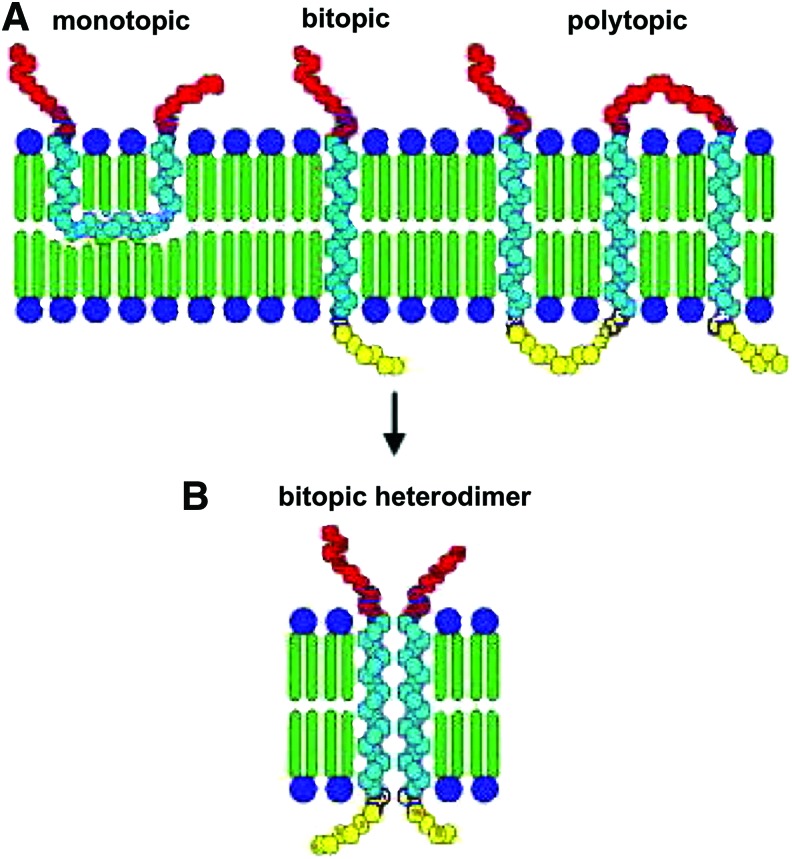
Configurations of α-helical peptides in an amphiphile bilayer. (**A**) Monotopic, bitopic, and polytopic. (**B**) Two different bitopic transmembrane peptides forming a functional heterodimer. Color graphics available at www.liebertonline.com/ast

Abiotically synthesized α*-*helical peptides in the range of 10-mers could be hosted within and stabilize primitive lipid bilayers approximately half the length of those in cell membranes, which readily assemble from acyl chains such as decanoic acid. Depending on their length, these primitive peptides can form bitopic (and possibly monotopic or, less likely, polytopic; Fig. 5A) configurations in prebiotic amphiphile bilayer vesicles. Bitopic helix oligomerization is a spontaneous process because it decreases the number of helix-lipid interactions to the more thermodynamically favored increase in the number of lipid-lipid interactions (Arkin, 2002). The formation of primitive bitopic transmembrane α-helices is crucial for the origin of life because the process of bitopic α-helix oligomerization (*e.g.,* two different bitopic transmembrane peptides joined in a homo/heterodimer; Fig. 5B) is known to transform a simple membrane peptide anchor into a biologically active complex (Arkin, 2002). Moreover, bilayer hydrocarbon acyl chain length is known to enhance activity, as in protein transporters for chain lengths spanning from 12 to 24 carbon atoms (Andersen and Koeppe, 2007).

Therefore, while peptides will spontaneously start forming conglomerates with other transmembrane peptides [of preferred antiparallel arrangement (Nir and Barry, 1996)], they will acquire various catalytic activities due to their overhanging/protruding polar termini; the most stable helix conformation is when both termini are protruding (Nir and Barry, 1996) since alone they are not functional. They would possibly act as initial ion gradient generators of energy and catalytic sites, membrane-signaling receptors, sites for primitive cytoskeleton anchoring and for serving the metabolic needs of primitive protocells. This is supported by the fact that di-, tripeptides made with combinations of Ala, Val, and Ser, and possibly ancient ones too, can catalyze the direct asymmetric intermolecular aldol reaction, which is fundamental in organic synthesis (Zou *et al.,* 2005). Moreover, weathering of minerals like apatite could have provided phosphate groups required for the formation of the high-energy phosphate-phosphate anhydrite bonds (Banfield *et al.,* 1999; Rogers and Bennett, 2004; Röling *et al.,* 2015).

This process of bitopic peptide functional acquisition can help answer how primitive transporters for ion gradient generation were first formed and how they can be functionally diversified. For example, bitopic peptide or oligomeric complex functional transformation to high-energy proton pumps could take place by subsequent binding of pigments such as ferrous iron and iron complexes with organic compounds like polycyclic aromatic hydrocarbons and their derivatives (Deamer, 1997, 2007).

There is evidence that a limited number of small peptide- and protein-forming amino acids may have existed 4 billion years ago (Longo *et al.,* 2013) and can be easily formed abiogenically (Rodriguez-Garcia *et al.,* 2015). Single-spanning (or bitopic) proteins are known to serve many functions in Earth life, given the fact that they represent more than half of all membrane protein in analyzed genomes; for example, in bacteria 41% of bitopic proteins are transporters (Hubert *et al.,* 2010). Some of these peptides can form pores that are highly selective. Examples of oligomerized bitopic transmembrane transporter peptides are the M2 proton channel of the primitive viral life-form Influenza B, a noncovalent tetra homo-oligomer of a small bitopic membrane protein in which the ion channel activity is entirely due to the transmembrane α-helical bundle, and the cell surface receptors tyrosine kinases, human glycophorin A and phospholamban (Arkin, 2002). Another example is gramicidin, which forms ion-conducting pores (Burkhart *et al.,* 1999).

In addition, the propensity of meteoritic amino acids to form α-helices even in aqueous/charged environments (Table 2) can be very important for the development of primitive protein-RNA complexes with ribosome-like functions that are required for translating stored genetic information. For example, eukaryotic 80S ribosomes contain nascent chains with high α-helical content located within the exit tunnel. Particularly interesting is the 6-turn α-helix located in the lower tunnel region near the exit because it has the length of a membrane bilayer (30 Å length) and assists in membrane insertion and/or promotes the correct folding of cytoplasmic proteins (Bhushan *et al.,* 2010). The ribosomal 50S subunit protein L9 in prokaryotes (*e.g., Salmonella enterca* and *Bacillus stearothermophilus*) is an elongated protein with a 9-turn α-helix, similar in structure to biopic transmembrane ones, which connects its functional N- and C-terminal globular domains and fixes their relative separation and orientation within the ribosome (Hoffman *et al.,* 1994; Leipuviene and Björk, 2007). This is consistent with the fact that the abiogenic amino acids are code-diversified by evolutionary mutations, possibly because they were available for longer times than the invented ones. This is supported by the fact that these have the highest genetic code redundancy [Gly (4), Ala (4), Val (4), Leu (6), Ile (3), Thr (4), Ser (6), Pro (4), Asp (2), Glu (2); number of codons in parentheses] compared to the invented ones [they have genetic code redundancy 1–2, with the exception of Arg (5)].

Membranes can be viewed as the first sites of highly concentrated catalytic centers via the immobilization of the transmembrane oligopeptide conglomerates, which is required for the primitive metabolism of protocells to be operational. For catalysis to take place, the organocatalysts must be at high densities that maximize reaction rates. The biochemistry of Earth life requires cells to be densely packed with proteins. For instance, they comprise ∼50% of a cell's dry weight (Watson, 1972) and 20% of its volume because such density maximizes biochemical reaction rates. At lower densities, proteins collide too rarely, while at higher densities, proteins diffuse too slowly through the crowded cell (Dill *et al.,* 2011). Protocells may have achieved initial high densities of proteins in the form of primitive peptides, bitopic or other conglomerate types of hydrophobic organocatalysts, by their spontaneously partitioning into lipid bilayers. Such protein immobilization is represented in Earth life by the densely packed membrane electron transport chains in bacteria, mitochondria, and chloroplasts.

Thus, *membrane catalyst density is also likely to be a universal property for the biochemistry of extraterrestrial life*.

### 2.6. Amino acid propensities in extremophilic proteins

Extraterrestrial life in our solar system, extinct/extant, would be more likely to exist as microorganisms developing under extreme environmental conditions. If such life is made of proteins with α-amino acids similar to Earth's life, these would have been selected for protein structure adaptation similar to those for Earth extremophiles. These thrive in temperature ranges from 0°C to 120°C, pH from 0 to 11, saturated salt solutions, and even in the water core of nuclear reactors. While it is believed that the last common ancestor to Earth life was a thermophile that passed its properties to the existing hyperthermophiles, it is also postulated that life may have originated in cold environments (Bada *et al.,* 1994). Thus, cold-adapted extremophiles may provide insight into the search for extraterrestrial life on Mars (deep or near the surface) and moons such as Europa (Levy *et al.,* 2000; Cavicchioli, 2002). For the latter, heat- and salt-adapted extremophiles may also be used as a yardstick, since it has been postulated that hydrothermal plumes develop after the initial period of heating, differentiation, and transport of heat and salt from Europa's silicate mantle to its ice shell (Travis *et al.,* 2012). It has also been suggested that salt solutions (approximated by the MgSO_4_ composition of brine from Eddy County, New Mexico) existed at one time on Mars, and presumably they became more concentrated as ice formed, thus providing an environment for the growth of halophiles (Litchfield, 1998).

Earth extremophiles include representatives of all three domains (Bacteria, Archaea, and Eukarya). However, the majority are microorganisms, and a high proportion of these are archaea (Cavicchioli, 2002). Thus, it is of particular interest to explore the amino acid compositions of archaean extremophiles, particularly the thermophiles, psychrophiles, and halophiles. Other types of extremophiles such as acidophiles, alkaliphiles, and piezophiles (thermophilic or psychrophilic deep in the oceans) have less obvious adaptations toward pH and pressure (Reed *et al.,* 2013).

Amino acid propensities reflected in extremophile protein adaptations are discussed in the following sections, and distinct biomarkers for extraterrestrial life are proposed based on certain amino acid propensities observed in terrestrial extremophiles. Biomarkers for extremophilic amino acid patterns are analyzed in terms of polarity, charge, and hydrophobicity (Table 3). As such, they could serve as complementary to the biomarkers already discussed in the preceding sections.

**Table 3.** Propensities of Amino Acids for Extremophilic Proteins^a^

*Thermophilic*	*Psychrophilic*	*Halophilic*
Polar acidic vs. uncharged aa:	Polar uncharged vs. acidic aa:	Polar acidic vs. basic aa:
[Arg + Glu] >	[Thr + Asn + Gln] >	[Glu + Asp] >
> [Asn^b^ + Gln^b^]	> [Arg + Glu]	> [Lys + Arg + His] ( = 1.4–2.3)
Within polar uncharged aa:	Within hph to borderline hph aa:	Within hph to borderline hph aa:
[Ser + Thr + Tyr] >	[Gly + Ala + Val] >	[Gly + Ala + Val] >
> [Asn^b^ + Gln^b^ + Met^b^]	> Leu^c^ + Ile^c^ + Phe + Met + Trp]	> Leu^c^ + Ile^c^ + Phe + Met + Trp]
Increase of hph aa:	Decrease of hph aa:	Decrease of hph aa:
Gly, Ala, Val, Leu, Ile, Phe, Pro	Gly, Ala, Val, Leu, Ile, Phe, Pro	Gly, Ala, Val, Leu, Ile, Phe, Pro
Polar charged > uncharged aa:		Within polar uncharged aa:
[Lys + Arg + His + Asp + Glu] >		[Thr + Cys] > [Ser]
> [Ser + Thr + Asn + Gln + Cys]		

aa = amino acids. hph = hydrophobic.

^a^ Table is composed from the data presented in the text.

^b^ Thermolabile amino acids.

^c^ Having medium-sized side-chains. The 20 amino acids are classified as follows: *Polar charged (basic):* Lys and Arg (both very basic), His (uncharged, or positively charged at pH ∼7); *polar charged (acidic):* Asp, Glu; *polar uncharged:* Ser and Thr (both with group -OH), Cys (-SH), Asn and Gln (both having group O = C-NH__2__); *nonpolar (hydrophobic):* Gly, Ala, Val, Leu, Ile, Pro, Phe, Met; *large hydrophobic:* Val, Ile, Leu, Met, Phe (highest the last two); *aromatic:* Phe, [Trp (indole group), Tyr (-OH group)] (both are borderline hydrophobic).

#### 2.6.1. Propensities for thermophilic proteins

Several factors are implicated in thermal stabilization of proteins and enzymes, and may differ in thermophilic and mesophilic organisms. The reasons for these differences are not yet fully known but may involve ratios of charged versus uncharged amino acids, hydrophobicity, ionic interactions, codon usage, and protein surface area (Kumwenda *et al.,* 2013). Other factors include varying amino acid flexibilities, or loop deletions, differences in disulfide bonds (Dill *et al.,* 2011), and Cys clustering in thermophilic proteomes (Rosato *et al.,* 2002). There may also be an increase in the number of ion pairs with higher growth temperature (Szilágyi and Závodszky, 2000). Other studies conclude that both thermophilic and mesophilic proteins have similar hydrophobicities, compactness, oligomeric states, polar and nonpolar contribution to surface areas, main-chain and side-chain hydrogen bonds, insertions/deletions and proline substitutions (Kumar *et al.,* 2000).

Compared to mesophilic proteins, the change of amino acid composition in thermophilic proteins can be categorized under the following distinctive trends (Zhou *et al.,* 2008; Reed *et al.,* 2013): (i) increase in nonpolar amino acids, especially the hydrophobic ones and Pro, which contribute to the hydrophobic interactions; (ii) increase in polar charged amino acids, especially Arg and Glu, which contribute to the ionic interactions; (iii) increase in aromatic amino acids, especially of Tyr, which contribute to the cation-π interactions and also to hydrophobic interactions; (iv) decrease in Met and polar uncharged Asn and Gln, as they are thermolabile; (v) replacement of polar uncharged surface residues with polar charged residues; (vi) increase in the number of disulfide bonds.

These general trends are corroborated by other studies which show the following: Gly, Ser, Lys, and Asp in mesophiles are generally substituted by Ala, Thr, Arg, and Glu, respectively (Argos *et al.,* 1979; Zhou *et al.,* 2008). High-throughput comparative analysis of complete proteomes shows extremely strong bias toward Arg-to-Lys replacement in hyperthermophilic organisms and overall Lys > Arg in hyperthermophiles (Berezovsky *et al.,* 2005). Amino acid compositions in extremely thermophilic proteins lead to an increase in the percentages of all charged residues including Lys and to a decrease in Met and Asn due to the chemical instability of these residues at high temperature (Szilágyi and Závodszky, 2000).

#### 2.6.2. Propensities for psychrophilic proteins

Psychrophiles, in general, are a class of extremophiles that grow at temperatures below 20°C and as low as 0°C in the liquid water films surrounding ice crystals. For psychrophilic proteins to maintain high activity at low temperatures, they must be able to move and change conformation due to a more flexible structure. This requires weak protein interactions, lower thermal stability, and increased specific activity attained by enlarged substrate binding area and poor affinity for the active site (Reed *et al.,* 2013). The adaptations of the psychrophilic proteins in archaean psychrophiles (Saunders *et al.,* 2003; Feller, 2010; Reed *et al.,* 2013) are summarized as follows: (i) increase of Gly residues, which provide greater conformational mobility in psychrophilic proteins; (ii) decrease of Pro residues in the loop regions (more Pro residues provide higher conformational rigidity); (iii) decrease of Glu and Arg as to decrease salt bridge and hydrogen bond formation; (iv) decrease in hydrophobic amino acids, particularly the large ones (Leu, Trp) in the protein core (to create weaker hydrophobic interactions); (v) increase of polar uncharged amino acids (particularly Gln and Thr).

#### 2.6.3. Propensities for halophilic proteins

Organisms that thrive in extremely salty environments have two major adaptations to extremely high salt concentrations. Some halophiles, mostly halophilic bacteria and eukaryotes, are protected against high concentrations of inorganic salts (*e.g.,* NaCl) by synthesizing osmolytes (small organic molecules like ectoine) that balance the osmotic pressure. Halophilic archaea, though, survive even with high intracellular concentrations of inorganic salts, which requires adaptations that allow their proteins to remain stable and functional (Reed *et al.,* 2013). The adaptations in the psychrophilic proteins of archaean halophiles are summarized as follows:

(i) Increased acidic residues like Glu and Asp on the protein surface (allows the protein to remain soluble by competing with ions for water), with concomitant decrease of the basic residues. In the aerobic halophilic archaea *Halobacter* and *Salinibacter*, the ratio [Glu + Asp]/[Lys + Arg + His] is 1.42–1.26 (Oren, 2013); in the halophilic Euryarchaeota (*e.g., Haloarcula marismortui, Natronomonas pharaonis, Salinibacter ruber*), the average ratio of [% negatively]/[% positively] charged amino acids is 2.3 versus 2.6 for the nonhalophilic (*i.e.,* ∼15% higher) (Paul *et al.,* 2008); and in halophilic strains, in general, the negatively charged amino acid residues are 18.5% higher compared to their nonhalophilic counterparts (Ebrahimie *et al.,* 2011).
(ii) Decreased Ser residues (effective for interaction with water but not for competition with charged ions).
(iii) Decreased number of hydrophobic residues, and replacement of the large and the aromatic (Phe) amino acids with smaller ones (Ile is replaced with Val) as to reduce the hydrophobic interactions (Siglioccolo *et al.,* 2011; Zhang and Ge, 2013).
(iv) Significant increase in the uncharged polar Thr (it is borderline hydrophobic) and Cys residues (Paul *et al.,* 2008).

## 3. Methods for Measuring Functional Properties of Amino Acids and Other Organic Compounds

I will conclude this study by summarizing technological developments that could be adapted for incorporation into instrument packages designed to monitor catalytic groups and amino acid patterns in life-detections missions.

Detection of amino acids and their catalytic groups
*The MOMA instrument.* It can identify amino acids in the martian subsurface (Goesmann *et al.,* 2017).
*Antibody technology.* Amino acid catalytic group antibodies should be immobilized on inorganic/organic supports to avoid cross-reactivity. This methodology can use commercial antibodies for the following detections:
Imidazole groups
Imidazole ELISA Kit (KA1421): Competitive binding enzyme immunoassay for the photometric quantitative measurement of imidazole. It cross-reacts (∼45%) with the small-sized histamine (as it also carries imidazole) but not much with the larger His. Available from Abnova (http://www.abnova.com/products/products_detail.asp?catalog_id=KA1421) and Novus Biologicals (http://www.novusbio.com/elisa-kits/imidazole).
Anti-Zilpaterol (N-Imidazol) antibody: Available from Antibodies-Online (http://www.biocompare.com/pfu/110447/soids/194761/Antibodies/Imidazole).
*His (imidazole group).* His tag monoclonal antibodies (by GenScript), and monoclonal anti-polyHistidine antibody (by Sigma).
Arg (guanidinium group)
Anti-Arginine antibody: Available from Abcam (http://www.abcam.com/arginine-antibody-ab48586.html).
Anti- L-Arginine Antibody Products: Available from Biocompare (http://www.biocompare.com/pfu/110447/soids/213742/Antibodies/L-Arginine).
*Gln (amide group).* Anti-Glutamine antibody: Available from Abcam (http://www.abcam.com/glutamine-antibody-ab9445.html).
*Nanopore-based techniques.* Nanopore-based instruments have already been proposed as biosensors for planetary missions, and the related technology has been reviewed (Rezzonico, 2014). Amino acids can be quantified by specific nanocomposite sensors based on various detection principles (Yang *et al.,* 2015). Indicatively, these can be based on molecularly imprinted photonic films (Xu *et al.,* 2012) and/or based on one-dimensional nanostructures of similar sensitivities (Feigel *et al.,* 2011), with sensitivity reaching 1 f*M* (Hu *et al.,* 2008). For example, Cys (and homo-Cys) can be detected (at 40 μ*M*) by a CuO/ZnO nanocomposite rod after induction of a blue shift in the rod's absorption spectra from 725 to 650 nm (due to the reduction of Cu^2+^ to Cu^0^ and subsequent oxidation of Cys to a disulfide bond) (Šimšíková *et al.,* 2014). Prospective technologies can be developed for amino acids as well, using, for example, carbon-nanomaterial electrochemical sensors (Tiwari *et al.,* 2016) and vertically aligned carbon nanofiber nanoelectrode arrays (Gupta *et al.,* 2014a).
*Detection of the catalytic (hydrolytic) activity of organic matter.* Samples of extraterrestrial organic matter are tested for hydrolytic activity by incubation with various nonfluorescent artificial substrates tagged with a fluorophore (Badalassi *et al.,* 2000; Wahler *et al.,* 2001; Albada and Liskamp, 2008). The identification of the catalytic function of the sample is made from the fluorescence of the released substrate fluorophore when a matching substrate/catalyst combination is met. The fluorophore could be detected, for example, by adaptation of an instrument based on an optofluidic chip (Yin *et al.,* 2006, 2007; Rudenko *et al.,* 2009). Screening for hydrolase (*e.g.,* esterase, lipase, amidase) activity can be performed with the periodate-coupled fluorogenic assay, using various artificial substrates tagged with the fluorescent probe umbelliferone, or with other chromophores such as the fluorescent 6-methoxynaphthaldehyde and the fluorescent/absorbing *p*-nitrophenol (Badalassi *et al.,* 2000; Wahler *et al.,* 2001). For esterase activity, the artificial substrate 7-acetoxycoumarin can be also used, which upon hydrolysis releases the fluorescent 7-hydroxycoumarin (Albada and Liskamp, 2008).
*Oligopeptide propensity for α-helicity*
  (A)  Identification of the high propensity in intramembrane α-helicity amino acids Ile, Leu, Val, Ala can be performed by, for example, carbon nanotube sensors with selective affinity for peptides (Wang *et al.,* 2003), with an example being the label-free detection of C-reactive protein by a carbon nanofiber-based biosensor (Gupta *et al.,* 2014b).
  (B)  Ile, Leu, Val, Ala, as well as Phe, Tyr, Trp can be identified as free residues by the MOMA instrument in the martian subsurface soil.
  (C)  Phe, Tyr, Trp can also be identified on martian topsoil and in the plumes of Europa and Enceladus by their intrinsic fluorescence (Smith *et al.,* 2014).

## Notes

^1^ These are the 50 abiogenic possibly prebiotic amino acids (included are the encoded Gly, Ala, Val, Pro, Glu, Asp, Leu, and Ile) identified in the Murchison meteorite, plus the 9 invented amino acids and the 14 intermediates of the metabolic pathways that terrestrial life uses for amino acid synthesis.
